# Case Report: Allogeneic hematopoietic stem cell transplantation in a patient with triple-allele expression at both HLA-B and HLA-C loci

**DOI:** 10.3389/fimmu.2026.1785227

**Published:** 2026-03-04

**Authors:** Yoshiyuki Fujita, Kyoko Yoshihara, Daisuke Takahashi, Hidenori Tanaka, Yusuke Kino, Fuyuki Yamagata, Mami Samori, Saki Takahashi, Satoshi Yoshihara

**Affiliations:** 1 Department of Hematology, Hyogo Medical University, Nishinomiya, Japan; 2 Department of Transfusion Medicine and Cellular Therapy, Hyogo Medical University Hospital, Nishinomiya, Japan; 3 Japanese Red Cross Society Blood Service Headquarters Central Blood Research Institute, Tokyo, Japan; 4 HLA Foundation Laboratory, Kyoto, Japan

**Keywords:** allogeneic hematopoietic stem cell transplantation, alloimmunity, donor selection, HLA duplication, HLA-B, HLA-C, triple alleles

## Abstract

**Background:**

Human leukocyte antigen (HLA) matching is critical for donor selection in allogeneic hematopoietic stem cell transplantation (allo-HSCT). Each HLA locus is normally biallelic; however, exceptionally rare cases with more than two alleles at a single locus have been reported.

**Case presentation:**

We describe a 17-year-old male with mixed phenotype acute leukemia (T/myeloid) who was found to carry three alleles at both the HLA-B and HLA-C loci during pre-transplant evaluation. Family-based HLA typing revealed that the additional alleles were inherited as part of a single maternal haplotype shared by multiple siblings. Flow cytometric analysis using antisera with known HLA specificity demonstrated that all three HLA-B and HLA-C alleles were expressed on the cell surface. Because this unusual immunogenetic configuration precluded the identification of an unrelated donor, a sibling donor sharing the same triple-allele haplotype was selected despite a single HLA-A mismatch. Allo-HSCT was successfully performed, with manageable graft-versus-host disease.

**Conclusion:**

This case highlights an extremely rare immunogenetic configuration in which triple-allele expression at two HLA class I loci directly influenced donor selection for allo-HSCT. Comprehensive interpretation of HLA typing results, including family analysis and protein-level expression, is essential when such atypical findings are encountered.

## Introduction

High-resolution donor–recipient matching of human leukocyte antigen (HLA) loci is a cornerstone of successful allogeneic hematopoietic stem cell transplantation (allo-HSCT) (1). In humans, each HLA locus is typically represented by two alleles inherited from the paternal and maternal haplotypes. Advances in molecular HLA typing have revealed rare structural variations within the HLA region, including gene duplications and copy number variations, which may result in the detection of more than two alleles at a single locus (2).

Reports of individuals carrying three alleles at a single HLA locus are exceedingly rare, and the clinical implications of such findings—particularly in the context of allo-HSCT—remain poorly defined. Here, we report a patient with tri-allelic expression at both the HLA-B and HLA-C loci, in whom donor selection for allo-HSCT was directly constrained by this immunogenetic configuration.

## Clinical course

A 17-year-old male presented with cervical lymphadenopathy and marked leukocytosis. Histopathological examination of lymph node and bone marrow specimens led to a diagnosis of mixed phenotype acute leukemia (MPAL) with T/myeloid features. Induction therapy with a pediatric acute lymphoblastic leukemia regimen was initiated but proved refractory. Subsequent salvage chemotherapy achieved a complete metabolic response, and allo-HSCT was planned due to the high risk of relapse.

## HLA typing and family analysis

**Figure 1:**
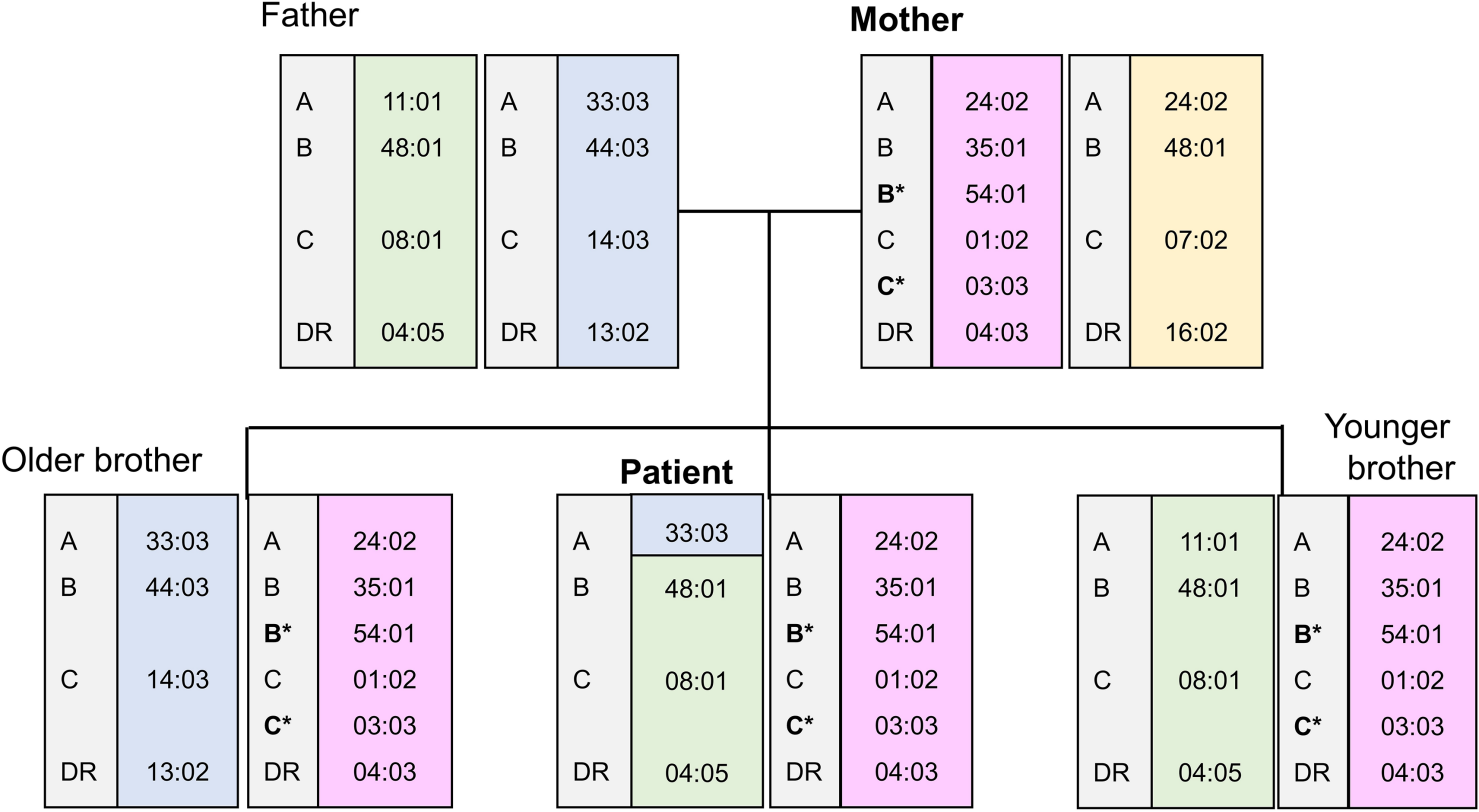
Family haplotype analysis demonstrating maternal inheritance of a haplotype carrying duplicated HLA-B and HLA-C alleles.

High- to intermediate-resolution HLA typing using a PCR-SSOP Luminex-based assay revealed the presence of three distinct alleles at both the HLA-B and HLA-C loci in the patient. Conventional cytogenetic analysis using G-banding demonstrated a normal karyotype, with no evidence of numerical or structural chromosomal abnormalities, including partial trisomy of chromosome 6. To rule out technical artifacts and to clarify the segregation pattern, HLA typing was additionally performed in his mother, and two siblings (**Table 1**). Although the father’s HLA testing was not available, the inheritance of the HLA haplotype, as shown in **Figure 1**, was inferred from the results of the patient, mother, and two siblings. The patient’s mother and two siblings also exhibited three alleles at both loci, indicating that the tri-allelic configuration was inherited as part of a single maternal haplotype and was stably transmitted to multiple offspring.

**Table 1:** HLA typing results showing three alleles at the HLA-B and HLA-C loci detected by PCR-SSOP Luminex analysis.

	HLA-A		HLA-B			HLA-C			HLA-DRB1	
Patient	24:02	11:01	35:01	54:01	48:01	01:02	03:03	08:01	04:03	04:05
Mother	24:02		35:01	54:01	67:01	01:02	03:03	07:02	04:03	16:02
Older brother	24:02	33:03	35:01	54:01	44:03	01:02	03:03	14:03	04:03	13:02
Younger brother	24:02	33:03	35:01	54:01	48:01	01:02	03:03	08:01	04:03	04:05

## Confirmation of HLA antigen expression

**Figure 2:**
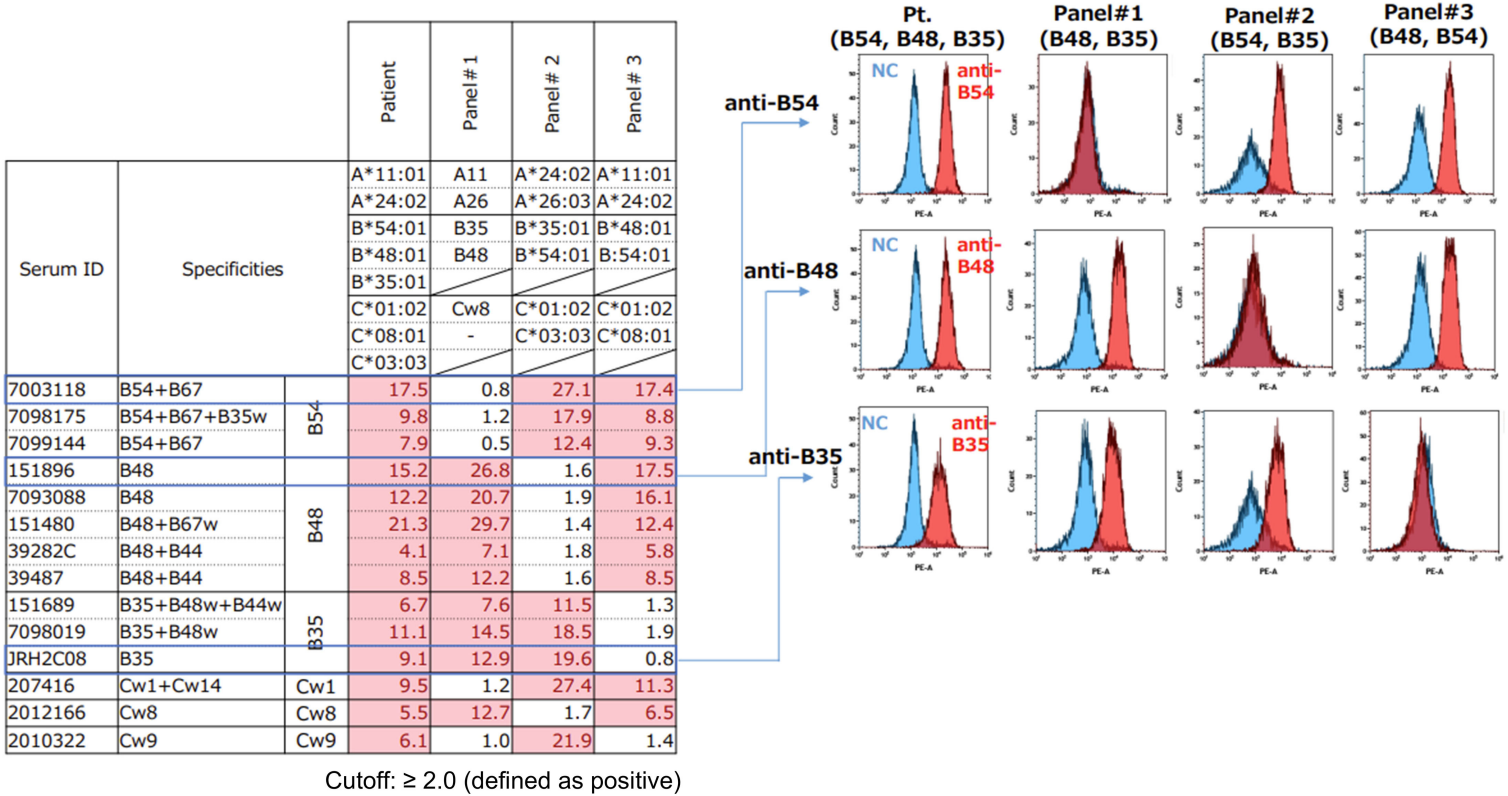
Flow cytometric analysis confirming surface expression of all three HLA-B and HLA-C antigens on patient lymphocytes. Representative staining with allele-specific antisera demonstrates distinct reactivity corresponding to each expressed HLA-B and HLA-C antigen, as compared with control panel cells lacking the respective specificities.

To determine whether all detected alleles were expressed on the cell surface, flow cytometric analysis was performed using antisera with defined HLA specificity (**Figure 2**). Peripheral blood lymphocytes of the patient were incubated with allele-specific anti-HLA antisera, followed by staining PE-conjugated anti-human IgG secondary antibodies. Surface expression of HLA antigens was analyzed by flow cytometry using appropriate controls. The results demonstrated clear and reproducible surface expression of all three HLA-B and HLA-C antigens.

## Donor selection and transplantation

Because identification of a fully matched unrelated donor was not feasible, a younger sibling was selected as the donor because he shared the same maternal haplotype carrying duplicated alleles at the HLA-B and HLA-C loci, as well as the inferred paternal haplotype, with the patient. There was a single mismatch at the HLA-A locus on the paternal haplotype, presumably due to recombination between the HLA-C and HLA-A loci (**Figure 1**). Peripheral blood stem cell transplantation was performed following a myeloablative conditioning regimen consisting of etoposide (30 mg/kg), cyclophosphamide (120 mg/kg), total body irradiation (12 Gy), and thymoglobulin (2 mg/kg). For graft-versus-host disease (GVHD) prophylaxis, tacrolimus, mycophenolate mofetil (30 mg/kg/day), and methylprednisolone (1 mg/kg/day) were administered. A total of 6.25 × 10^6^ CD34^+^ cells/kg were infused. Neutrophil engraftment was achieved on day 10 post-transplant.

The patient developed severe infectious complications and acute cardiac failure in the early post-transplant period, requiring temporary mechanical ventilation. Subsequently, acute GVHD progressed to a maximum grade of III. This was managed with high-dose corticosteroid pulse therapy and additional treatment with bone marrow–derived mesenchymal stromal cells, resulting in improvement of GVHD. The patient was discharged on day 66 post-transplant but later experienced relapse of mixed phenotype acute leukemia and died 529 days after transplantation.

## Discussion

The present case demonstrates an exceptionally rare immunogenetic configuration characterized by tri-allelic expression at both the HLA-B and HLA-C loci, with direct clinical consequences for donor selection in allogeneic hematopoietic stem cell transplantation. Several lines of evidence support a genuine biological basis for this finding rather than a technical artifact, including reproducible detection across multiple family members, stable inheritance of the additional alleles within a single maternal haplotype, and confirmation of protein-level expression of all antigens by flow cytometry. Collectively, these findings are most consistent with structural variation within the HLA class I region, such as segmental gene duplication, rather than mosaicism or assay-related error (3, 4). To date, only a limited number of cases describing more than two alleles at a single HLA locus have been reported, involving both HLA class I and class II genes, and most were identified in the context of genetic or serological investigations rather than clinical transplantation (5, 6). Among these rare reports, distinct biological mechanisms have been implicated. Structural variations involving chromosome 6p, including partial trisomy, have been shown to alter gene dosage within the HLA region and may result in atypical HLA expression patterns. In addition, tri-allelic expression of HLA genes has been described in rare settings such as constitutional chimerism, representing a biological mechanism distinct from a duplication-based mechanism suggested in the present case (7, 8).

From an immunological perspective, the expression of additional HLA class I molecules is not merely a genotyping anomaly. HLA class I molecules play central roles in both adaptive and innate immunity through antigen presentation to CD8^+^ T cells and inhibitory signaling to natural killer (NK) cells (9). The presence of additional, independently expressed HLA-B and HLA-C molecules effectively increases the repertoire of alloantigens that may be recognized by donor-derived immune cells. Therefore, triple-allele expression should be regarded as a biologically meaningful source of alloreactivity, rather than a benign polymorphism.

A particularly important implication of this case lies in the limitation of unrelated donor searches. Current donor registries and matching algorithms are predicated on the assumption of biallelic HLA expression at each locus. In patients with triple-allele expression, the conventional definition of “full matching” becomes untenable, rendering the identification of an unrelated donor effectively impossible. This highlights a structural limitation of existing donor selection frameworks when confronted with rare immunogenetic variants.

In this context, the selection of a related donor sharing the same triple-allele haplotype at the HLA-B and HLA-C loci was considered immunologically rational, even in the presence of a single mismatch at the HLA-A locus. The observed recombination at the HLA-A locus further underscores that the HLA region, while characterized by strong linkage disequilibrium, is not genetically immutable (10). Recombination events within this region, although rare, can complicate the interpretation of apparent HLA mismatches during donor selection.

Although the patient ultimately experienced disease relapse, this outcome does not diminish the immunogenetic significance of the case. Rather, it emphasizes that the primary contribution of this report lies in elucidating how rare HLA structural variations can constrain donor availability and necessitate individualized decision-making strategies. When atypical HLA typing results are encountered, comprehensive evaluation—including family studies and confirmation of antigen expression—should be strongly considered to guide optimal donor selection.

## Conclusion

Triple-allele expression at multiple HLA class I loci is exceedingly rare but can have profound implications for allo-HSCT donor selection. Careful evaluation of inheritance patterns and antigen expression is essential to inform clinical decision-making in such cases.

## Data Availability

The original contributions presented in the study are included in the article/supplementary material. Further inquiries can be directed to the corresponding author.
